# Cancer-Related Psychological Distress over the Past Decade: A Bibliometric Analysis of Research Trends, Hotspots, and Emerging Areas

**DOI:** 10.3390/healthcare14142195

**Published:** 2026-07-20

**Authors:** Linfeng Wang, Xiaonan Xu, Baojin Hua, Rui Liu

**Affiliations:** Guang’anmen Hospital, China Academy of Chinese Medical Sciences, No. 5, Beixiange, Xicheng District, Beijing 100053, China; azqll@aliyun.com (L.W.); la1305348832@163.com (X.X.)

**Keywords:** cancer, psychological distress, psychosocial factors, bibliometric, research trends

## Abstract

Background: Cancer-related psychological distress is a major concern in comprehensive oncology care because it substantially impairs patients’ quality of life and may adversely affect treatment adherence, outcomes, and prognosis. Over the past decade, research in this field has expanded rapidly; however, the overall knowledge structure, global research patterns, major contributors, and emerging hotspots remain insufficiently characterized. A bibliometric analysis is therefore needed to systematically map the development of cancer-related psychological distress research and identify evolving directions for future investigation. Methods: Publications related to cancer-related psychological distress published between 1 January 2015 and 31 December 2024 were retrieved from the Web of Science Core Collection and Scopus databases. The database searches were conducted on 10 March 2025. Bibliometric analyses were performed using VOSviewer (version 1.6.20), CiteSpace (version 6.3.R1), and the R package bibliometrix (version 5.3). Publication trends, country and institutional contributions, journal distribution, author collaboration networks, co-cited references, keyword co-occurrence, burst keywords, and thematic evolution were analyzed. Results: A total of 7063 publications were included in the bibliometric analysis, including 6162 articles and 901 reviews. Annual publication output increased from 465 publications in 2015 to 924 publications in 2024. The main contributing countries were the United States, Australia, China, Germany, and the United Kingdom. The United States ranked first in publication volume and total citations. Psycho-Oncology and Supportive Care in Cancer were the leading journals by publication volume and total citations. Major research themes included quality of life, psychological distress, depression, anxiety, breast cancer, distress screening, survivorship, and palliative care. Seven high-frequency keywords—“cancer,” “quality of life,” “psychological distress,” “depression,” “anxiety,” “breast cancer,” and “distress”—each appeared more than 500 times, representing the core research topics. Emerging keywords such as “informal caregivers,” “young adults,” “guidelines,” and “adult survivors” reflected increasing attention to caregiver support, age-specific psychosocial needs, survivorship care, and standardized clinical management. Conclusions: This bibliometric analysis provides a comprehensive overview of global research on cancer-related psychological distress from 2015 to 2024. The findings reveal publication trends, major contributors, collaboration patterns, core research themes, knowledge structures, and emerging topics in this field. Current research has gradually shifted from general descriptions of psychological distress toward survivorship care, caregiver support, standardized screening, and guideline-based management. Future studies should strengthen interdisciplinary collaboration, improve standardized assessment and screening approaches, and further explore emerging areas such as caregiver support, young adult cancer populations, survivorship care, and digital health and artificial intelligence-assisted approaches, which require further validation before routine clinical implementation.

## 1. Introduction

Cancer remains a major global public health challenge and is one of the leading causes of death and morbidity worldwide. With ongoing population growth and aging, its global burden is expected to increase further. It has been projected that the number of new cancer cases will exceed 35 million by 2050 [1]. In addition to its substantial physical burden, cancer exerts profound psychological effects on patients. A recent study reported that the prevalence of psychological distress among patients with cancer is approximately 33.16%, indicating that nearly one in three patients experiences clinically significant distress during the course of the disease [2]. According to the National Comprehensive Cancer Network, distress is a multifactorial and unpleasant experience involving psychological, social, spiritual, and physical dimensions that may compromise a patient’s ability to cope effectively with cancer and its treatment [3]. As contemporary cancer care increasingly emphasizes quality of life and whole-course management, psychological distress has become a key issue in comprehensive oncology care [4].

Psychological distress in patients with cancer encompasses a wide range of manifestations, including anxiety, depression, fear, insomnia, helplessness, social withdrawal, and functional impairment [5]. Importantly, such distress may occur throughout the entire cancer trajectory, spanning diagnosis, treatment, rehabilitation, survivorship, and end-of-life care [6]. Accumulating evidence suggests that the association between cancer and psychological distress is bidirectional. On the one hand, cancer diagnosis, treatment-related adverse events, symptom burden, financial strain, and disruption of social roles may precipitate or aggravate psychological distress [7]. On the other hand, persistent distress may adversely affect treatment adherence, functional status, and quality of life, and may even contribute to poor prognosis through behavioral and biological mechanisms [8,9,10]. Therefore, psychological distress should not be viewed merely as a secondary emotional response to cancer, but rather as a clinically meaningful factor that warrants structured attention in oncology practice.

In recent years, advances in precision medicine, psychosocial oncology, and integrative medicine have driven rapid growth in research on cancer-related psychological distress. This body of research has expanded to include epidemiology, risk factors, underlying mechanisms, screening and assessment tools, psychosocial care, survivorship management, caregiver support, and digital health management [11]. Nevertheless, the field remains highly interdisciplinary and heterogeneous, with research themes evolving rapidly over time. As a result, conventional reviews are often insufficient to comprehensively delineate the knowledge structure, research landscape, and developmental trends of this field. A bibliometric and visualized knowledge-mapping approach is therefore useful for identifying the intellectual framework of the literature, major contributors, collaboration patterns, core themes, and emerging directions.

Therefore, this bibliometric analysis aimed to comprehensively map the global research landscape of cancer-related psychological distress from 2015 to 2024. Specifically, we sought to: (1) identify publication trends and major contributors; (2) map collaboration networks, keyword clusters, and co-citation structures; and (3) detect research hotspots, thematic evolution, and emerging frontiers in this field.

## 2. Materials and Methods

### 2.1. Retrieval Strategy

This study employed a bibliometric approach to quantitatively and visually map the research landscape, hotspots, and emerging trends in cancer-related psychological distress. The dataset was retrieved from the Web of Science Core Collection (https://www.webofscience.com; accessed on 10 March 2025) and Scopus (https://www.scopus.com; accessed on 10 March 2025). The search period covered publications from 1 January 2015 to 31 December 2024. Detailed search strategies for both databases are provided in Supplementary Material S1 to ensure transparency and reproducibility.

The Web of Science Core Collection and Scopus were both searched on 10 March 2025. The search period covered publications from 1 January 2015 to 31 December 2024. Detailed search strategies for both databases are provided in Supplementary Material S1 to ensure transparency and reproducibility. The search strategy combined topic terms and free-text terms related to cancer and psychological distress.

### 2.2. Research Selection and Data Extraction

All retrieved records were imported into EndNote X9 for reference management and duplicate removal. Two researchers (LW and XX) independently screened the titles and abstracts of the remaining records according to the predefined eligibility criteria. Records that were not relevant to cancer-related psychological distress, were not articles or reviews, or had incomplete bibliographic information were excluded. Full texts were consulted only when eligibility could not be determined from the title, abstract, or bibliographic information. Any disagreements were resolved through discussion, and when necessary, a third reviewer (RL) made the final decision. The extracted bibliographic information included titles, publication years, authors, journals, countries/regions, institutions, keywords, references, citation counts, and Web of Science categories.

### 2.3. Data Analysis

After screening, records retrieved from the Web of Science Core Collection and Scopus were exported in BibTeX format and imported into R software (version 4.4.2) for data merging and deduplication. The R package bibliometrix (version 5.3) was used to convert records from both databases into a standardized bibliographic data frame. Records from WoSCC and Scopus were then merged in R, and duplicate records were identified primarily by DOI. For records without DOI or with inconsistent DOI information, potential duplicates were further checked using title, first author, publication year, and journal name. After automatic duplicate removal, the merged dataset was manually verified to minimize duplicate records caused by differences in metadata across databases.

The final deduplicated dataset was imported into CiteSpace (version 6.3.R1), VOSviewer (version 1.6.20), and the R package bibliometrix (version 5.3) for subsequent bibliometric analyses. Extracted bibliographic information included titles, publication years, authors, journals, countries/regions, institutions, keywords, references, citation counts, and Web of Science categories. Country attribution was based on the country information extracted from author affiliations in the bibliographic records. When a publication included authors from multiple countries, each contributing country was counted in the country-level analysis. Therefore, country-level document counts reflected multi-country contributions and should not be interpreted as mutually exclusive categories.

Citation counts were obtained from the source database records. For duplicate records indexed in both WoSCC and Scopus, WoSCC citation counts were retained to ensure consistency; for records retrieved only from Scopus, Scopus citation counts were used. Therefore, citation indicators in this study should be interpreted as database-derived citation counts rather than field-normalized citation metrics. The analyses focused on annual publication trends, author collaboration networks, contributions from countries and institutions, journal distribution, keyword co-occurrence, co-citation relationships, research hotspots, and emerging themes in cancer-related psychological distress. Journal impact factors and quartile rankings were obtained from the 2024 Journal Citation Reports (JCR).

## 3. Results

### 3.1. Literature Selection

**Figure 1:**
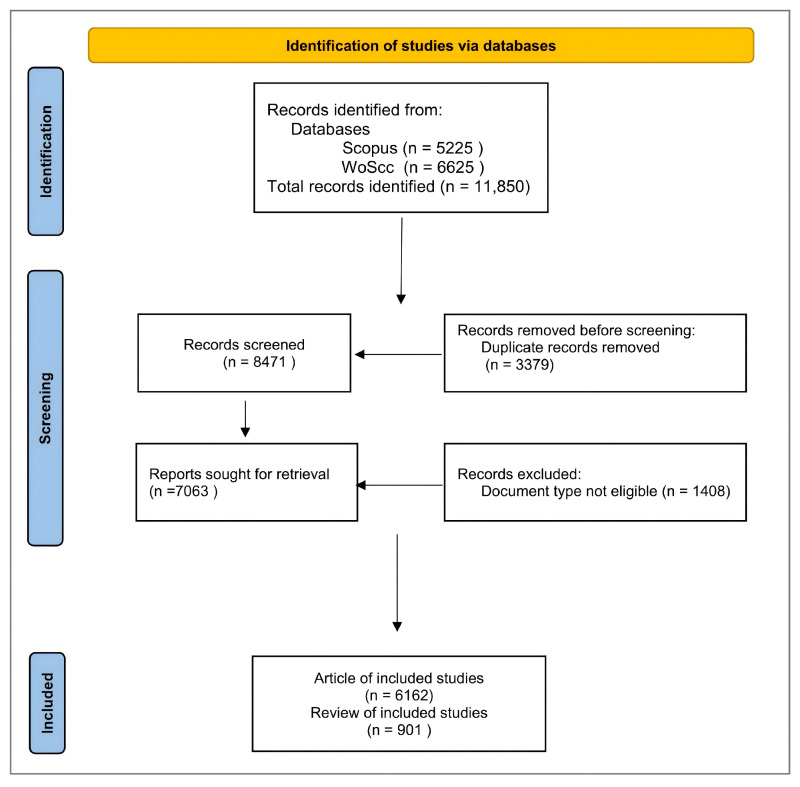
Flow diagram of record identification, screening, and inclusion for the bibliometric analysis.

A total of 11,850 records were initially identified from WoSCC and Scopus, including 6625 records from WoSCC and 5225 records from Scopus. After duplicate removal and screening according to the predefined eligibility criteria, 7063 publications were included in the final bibliometric analysis, including 6162 articles and 901 reviews. The process of record identification, screening, exclusion, and inclusion is shown in Figure 1.

### 3.2. Analysis of the Number of Publications

**Figure 2:**
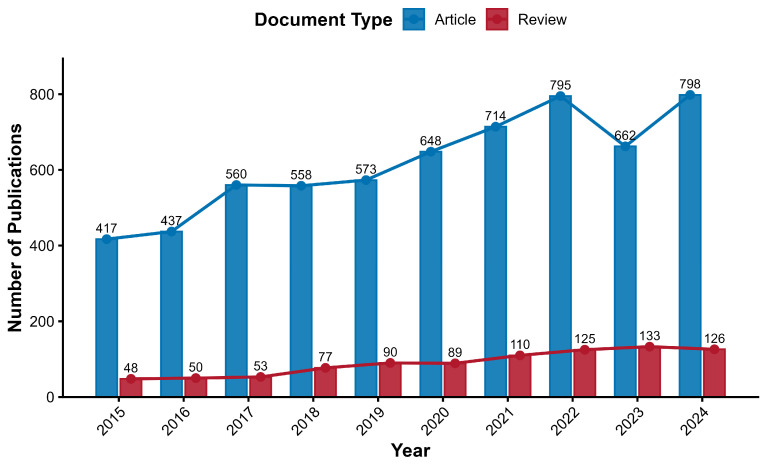
Annual distribution of publications on cancer-related psychological distress research from 2015 to 2024.

Figure 2 presents the annual distribution of publications by document type from 2015 to 2024. Overall, the annual number of publications showed a clear upward trend. The total publication output increased from 465 in 2015 to 924 in 2024, indicating a sustained expansion of research interest in this field. The growth pattern can be broadly divided into two stages. From 2015 to 2019, the number of publications increased steadily, reflecting a gradual rise in academic attention. From 2020 to 2024, publication output increased more markedly, with high levels observed in 2021, 2022, and 2024. Articles consistently accounted for the majority of publications each year, peaking at 798 in 2024, whereas reviews also increased over time, reaching the highest number in 2023.

### 3.3. Analysis of the Contributions of Countries

Country-level analysis showed that 108 countries/regions contributed to research on cancer-related psychological distress. The top 10 countries by publication volume are presented in Table 1. With regard to citations per paper, a metric calculated by dividing the total citations by the number of publications, the Netherlands ranks first among the top ten countries with a score of 38.0, suggesting a relatively high citation impact of its publications in this field. The other countries, listed in descending order of citations per paper, are Australia (31.0), the United Kingdom (30.2), Germany (26.8), Canada (26.3), the United States (25.8), Spain (20.1), Italy (19.1), China (16.1), and Japan (12.1). Although China ranks third in terms of publication volume (*n* = 650), its total citations place it seventh, and its average citations per paper place it ninth, indicating a difference between publication quantity and citation-based impact.

**Figure 3:**
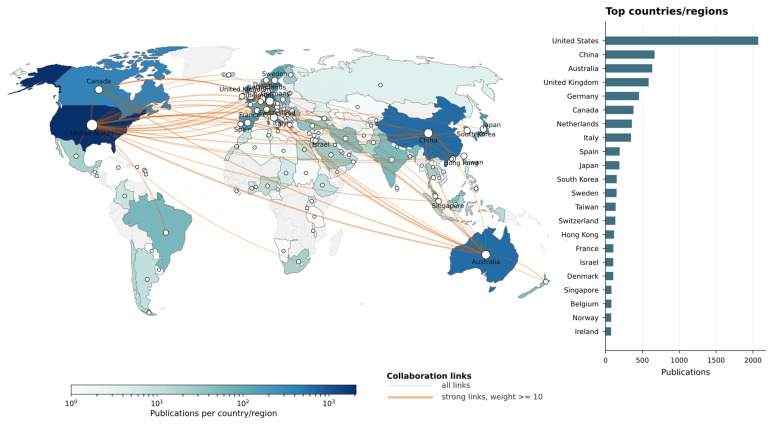
A map of country contribution and collaboration based on the article output. The size of each circle represents the number of publications from the corresponding country/region, with larger circles indicating higher publication output.

Figure 3 shows the country collaboration network. The United States had the highest total link strength and showed collaborative links with countries such as China, Australia, Germany, the United Kingdom, and Canada. Overall, the country-level results reveal differences in publication output, citation performance, and international collaboration patterns across countries.

### 3.4. Analysis of the Contributions by Institutions

Institutional analysis showed that 7181 institutions contributed to publications on cancer-related psychological distress. Table 2 presents the top 10 institutions ranked by publication volume, together with total citations, mean citations per publication, and total link strength. Among these institutions, six were located in the United States, two in Australia, one in Canada, and one in the Netherlands. The University of Sydney ranked first in publication volume and also showed the highest total link strength, indicating a prominent role in institutional collaboration. Harvard Medical School and the University of Melbourne also showed high publication output and strong collaborative connections. In terms of citation-based indicators, several institutions showed relatively high mean citation counts per publication, including the University of Sydney, the University of Toronto, Memorial Sloan Kettering Cancer Center, and Radboud University Nijmegen. These results indicate that institutional contribution varied across publication output, citation impact, and collaboration strength. These institutions appear to be important participants in transnational and cross-institutional research collaboration.

### 3.5. Journal and Co-Cited Journal Analysis

Table 3 and Table 4 present the top 10 journals in this field ranked by publication volume and citation count, respectively. *Psycho-Oncology* (483 publications, 13,078 citations) ranked first in both categories, followed by *Supportive Care in Cancer* (396 publications, 8056 citations). These findings suggest that these two journals have played central roles in publishing and disseminating research on cancer-related psychological distress.

Citation count reflects the extent to which a journal’s publications have been referenced within the academic community. In this respect, *Cancer* (111 publications, 5828 citations) also showed strong performance. It ranked highly in both publication volume and total citations, suggesting that it is an important publication venue in this field. In addition, although journals such as *Frontiers in Psychology* had lower citation counts than the top three journals, they remained active outlets for related research. In terms of citations per paper, the *Journal of Clinical Oncology* (CPP = 80.87) and *Cancer* (CPP = 52.50) performed better than the other journals listed, indicating relatively high citation impact for papers published in these journals. They were followed by the *Journal of Pain and Symptom Management* (CPP = 28.96), BMC *Cancer* (CPP = 28.35), *Psycho-Oncology* (CPP = 27.08), and *Supportive Care in Cancer* (CPP = 20.34). Among the ten journals with the highest publication volume, *Cancer* had the highest impact factor, and six journals were classified as JCR Q1. Among the ten most cited journals, the *Journal of Clinical Oncology* had the highest impact factor, and seven journals were classified as JCR Q1. Overall, the journal analysis suggests that research on cancer-related psychological distress has been published across both specialized psycho-oncology journals and broader oncology or supportive care journals, reflecting the interdisciplinary nature of this field.

### 3.6. Analysis of Author Impact and Collaboration

**Figure 4:**
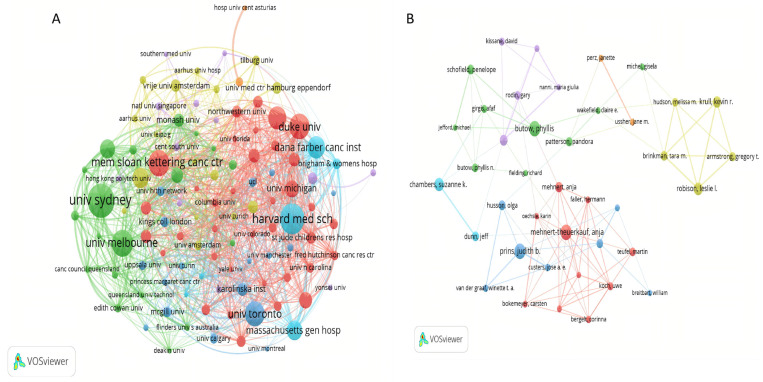
Institution and author collaboration networks. (**A**) Institutional collaboration network; (**B**) author collaboration network. Each node represents an institution or author, and the node size is proportional to the number of publications. Different colors indicate different collaboration clusters identified by VOSviewer. The connecting lines represent collaboration relationships, with thicker lines indicating stronger collaboration. All nodes are displayed as circles.

Author productivity and collaboration patterns are summarized in Table 5 and Figure 4B. After author name standardization, Anja Mehnert was the most productive author, with 58 publications and 1651 citations, followed by Areej El-Jawahri, Eduardo Bruera, Phyllis Butow, and Judith B. Prins. Table 6 presents the top 10 authors ranked by total citation count. Anja Mehnert ranked first in total citations, followed by Phyllis Butow, David Cella, Stephen Ross, and Areej El-Jawahri. In terms of mean citations per publication, Stephen Ross and David Cella showed particularly high values, although their publication volumes were lower than those of several other authors listed in the table. The author collaboration network revealed several relatively stable research clusters, with collaboration links mainly concentrated within specific author groups.

### 3.7. Reference Co-Citation Analysis

Reference co-citation analysis was performed to identify the intellectual base of research on cancer-related psychological distress. It should be noted that co-cited references may have been published before the 2015–2024 search period, because they represent references cited by the publications included in the bibliometric dataset rather than records directly retrieved in the search.

As Figure S1 illustrates, references with a co-citation frequency of at least 20 are displayed, among which the paper authored by Zigmond et al. in Acta Psychiatrica Scandinavica has garnered the highest number of citations (531), primarily concerning the utilization of anxiety and depression scales. The highly co-cited references are as follows: First, Donovan et al. (2014) in *Psycho-Oncology*, “Validation of the Distress Thermometer Worldwide: State of the Science” (141 citations). Second, Sung et al.’s “Global Cancer Statistics 2020: GLOBOCAN Estimates of Incidence and Mortality Worldwide for 36 Cancers in 185 Countries” (133 citations) provides a comprehensive analysis of global cancer statistics, offering valuable insights into the prevalence and mortality of various cancers worldwide. Another highly co-cited reference was the study by Mehnert et al. (2018), “One in Two Cancer Patients Is Significantly Distressed: Prevalence and Indicators of Distress,” published in *Psycho-Oncology*, which received 119 co-citations. Collectively, these highly cited publications are widely recognized as foundational references in this field.

### 3.8. Keywords Analysis

#### 3.8.1. Co-Occurrence Analysis

**Figure 5:**
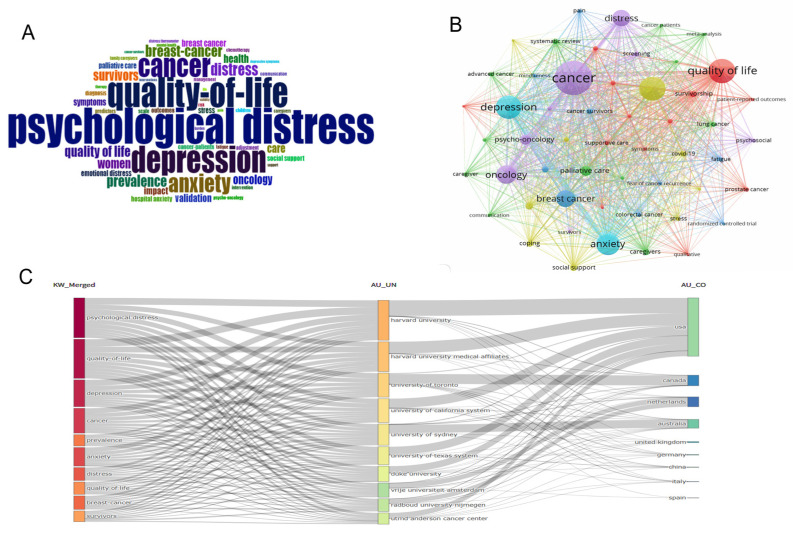
Keyword-related visualizations of research on cancer-related psychological distress. (**A**) Word cloud of high-frequency keywords. (**B**) Keyword co-occurrence network showing the major keyword clusters. Each node represents a keyword, and the node size is proportional to its frequency of occurrence. Different colors indicate different keyword clusters. The connecting lines represent co-occurrence relationships between keywords, with thicker lines indicating stronger associations. All nodes are displayed as circles. (**C**) Topic–institution–country/region relationship map showing associations among research topics, contributing institutions, and countries/regions.

Keyword co-occurrence analysis was performed to identify major research topics in cancer-related psychological distress. As shown in Figure 5A, the word cloud displays the most frequently occurring keywords, including “cancer,” “quality of life,” “psychological distress,” “depression,” “anxiety,” “oncology,” “breast cancer,” “distress,” “palliative care,” and “psycho-oncology.” These high-frequency keywords indicate that research in this field has mainly focused on psychological symptoms, quality of life, cancer care, and supportive care.

Figure 5B presents the keyword co-occurrence network. The network showed five major clusters, covering patient quality of life, cancer-related psychological symptoms and interventions, common cancer types associated with psychological distress, distress screening and early identification, and additional topics such as mindfulness, advanced cancer care, caregiver burden, and doctor–patient communication. Figure 5C further illustrates the relationships among major topics, institutions, and countries/regions, showing how research themes were connected with leading contributors and collaborative networks.

#### 3.8.2. Burst Analysis of Keywords

**Figure 6:**
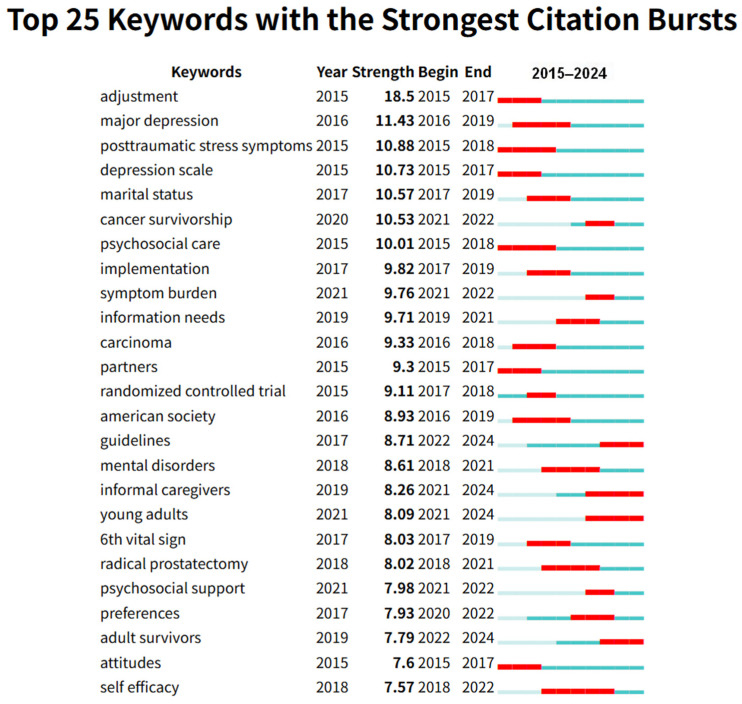
Top 25 burst keywords in research on cancer-related psychological distress from 2015 to 2024. The blue lines represent the overall study period, while the red lines indicate the periods during which the corresponding keywords exhibited citation bursts.

Keyword burst analysis was performed to identify terms that showed a rapid increase in occurrence frequency during specific periods from 2015 to 2024. Figure 6 presents the top 25 burst keywords. The early burst keywords included “adjustment,” “posttraumatic stress symptoms,” “depression scale,” “partner,” and “attitudes,” suggesting that earlier research attention was related to psychological adaptation, stress-related symptoms, assessment tools, and interpersonal factors.

Among the burst keywords, “self-efficacy” showed the longest burst duration, indicating sustained attention to patients’ perceived ability to cope with cancer-related challenges. In recent years, keywords such as “informal caregivers” (2021–2024), “young adults” (2021–2024), “guidelines” (2022–2024), and “adult survivors” (2022–2024) showed recent burst periods. These terms indicate increasing research attention to caregiver support, age-specific psychosocial needs, standardized clinical management, and survivorship-related psychological issues.

### 3.9. Analysis of Research Hotspots and Trends

The evolution of research hotspots provides insight into how the focus of studies within this field has shifted over time. As illustrated in Figure S2, the early hotspots (2015–2019) primarily encompassed metacognitive beliefs, self-rated health, unfavorable news, and hematological cancer, signifying the field’s initial phase of specialized exploration. Mid-term persistent hotspots (2019–2022) encompassed prostate cancer, psychosocial factors, oncology, depression, and anxiety, focusing on specific cancer types and their psychosocial dimensions. Long-term persistent hotspots during 2021–2024 include cancer-related fatigue, prehabilitation, barriers, digital health, and well-being, reflecting emerging and continuously prominent new directions in recent years.

## 4. Discussion

This bibliometric analysis provides an overview of the development, knowledge structure, and emerging directions of research on cancer-related psychological distress from 2015 to 2024. The steady increase in annual publications, especially after 2019, suggests that psychological distress has become an increasingly important topic in oncology, supportive care, and survivorship research. This trend reflects a broader shift in cancer care from a survival-centered model toward a more patient-centered approach that emphasizes quality of life, psychosocial adaptation, and long-term well-being.

The distribution of countries, institutions, journals, and authors indicates that research on cancer-related psychological distress has developed a recognizable academic structure, with publications concentrated mainly in psycho-oncology, supportive care, palliative care, nursing, psychology, and oncology journals. This pattern suggests that the field is no longer confined to a narrow psychosocial research area but has become increasingly integrated into mainstream oncology and supportive-care research. Country and institutional analyses further revealed substantial differences in research productivity, citation impact, and collaboration patterns. The United States ranked first in publication output, citation impact, and institutional participation, while the author and institutional collaboration networks showed several relatively stable research clusters. However, collaboration links between clusters remained limited, suggesting that future progress may depend on stronger cooperation across research teams, institutions, disciplines, and healthcare systems. Such collaboration may improve methodological consistency, support multicenter studies, and enhance the generalizability of findings across different clinical and cultural contexts. Nevertheless, the dominance of the United States and other highly productive countries should be interpreted cautiously, as bibliometric patterns may be affected by database coverage, language restrictions, citation practices, and indexing bias [12,13].

Keyword co-occurrence, clustering, burst detection, and thematic evolution analyses suggest that the field has moved from early attention to distress screening, symptom measurement, depression scales, and psychological adaptation toward more clinically oriented and translational themes. Three directions were particularly prominent: the integration of psychological distress with physical symptom burden and quality-of-life outcomes, increasing attention to adolescents and young adults with cancer, and the emergence of digital health and AI-assisted approaches. This transition suggests that research on cancer-related psychological distress is becoming less descriptive and more focused on practical strategies for screening, monitoring, intervention, and care integration.

The growing connection between psychological distress, quality of life, fatigue, symptom burden, palliative care, survivorship, and well-being indicates that distress is increasingly understood as part of a broader psychosocial–somatic symptom experience rather than as an isolated emotional problem. Psychological distress often coexists with pain, fatigue, sleep disturbance, nausea, reduced physical functioning, and other cancer-related symptoms, and these symptoms may interact with one another to impair functional status and overall well-being [14,15]. Within this trend, both pharmacological and non-pharmacological interventions have been explored as components of integrated supportive care. Previous studies have examined the potential role of psychotropic medications in selected cancer-related emotional and physical symptoms [16,17,18,19,20,21,22,23,24,25,26]. At the same time, non-pharmacological interventions remain an important component of integrated care. Evidence also suggests that psychological resilience is closely associated with emotional well-being and quality of life in patients with cancer [27]. Interventions such as behavioral activation therapy, music therapy, cognitive behavioral therapy, acceptance and commitment therapy, and multidisciplinary collaborative care have been used to reduce psychological distress and improve psychosocial outcomes [28,29,30]. Notably, the impact of cancer-related psychological distress is not limited to patients themselves, but also extends to caregivers and family members. Caregivers who receive insufficient support may also experience substantial psychological distress, which may further affect patient recovery and family functioning [31]. These findings suggest that future supportive-care models should move beyond patient-centered interventions alone and place greater emphasis on family-oriented, individualized, and multidisciplinary strategies. Overall, the growing attention to integrated psychosocial–somatic care reflects a shift in psycho-oncology from isolated symptom management toward comprehensive, patient-centered care. Pharmacological strategies may have a role in selected clinical contexts, but the overall trend in this field places greater emphasis on the integration of psychological support, physical symptom management, caregiver involvement, and multidisciplinary supportive care. Future research should further clarify applicable patient subgroups, treatment contexts, and outcome measures, and develop more standardized and mechanism-informed models to promote the effective integration of psychological care and somatic symptom management in oncology practice.

Another emerging hotspot identified in recent years is the growing focus on adolescents and young adults with cancer. This trend was reflected in the burst keyword “young adults,” suggesting that age-specific psychosocial needs have become an increasingly visible topic in cancer-related psychological distress research. Compared with older adults, adolescents and young adults often experience cancer during a critical period of education, career development, intimate relationships, fertility planning, identity formation, and financial independence. Disruptions in these life domains may intensify psychological distress and create long-term psychosocial consequences [32,33]. In addition, the increasing incidence of early-onset cancers in some populations may further contribute to the growing academic and clinical attention to this group [34,35].

The bibliometric emergence of “young adults” indicates that psychological distress research is moving from a general patient-centered framework toward more population-specific and life-course-oriented perspectives. Existing studies suggest that adolescents and young adults with cancer may face elevated risks of persistent distress, anxiety, depression, unmet supportive-care needs, and, in some high-risk groups, suicidal behavior [36,37,38]. However, in the context of the present bibliometric analysis, these findings should be used to explain why this population has become a research hotspot rather than to provide a comprehensive review of all psychological outcomes in young patients. The increasing attention to this group may reflect the recognition that psychological distress is shaped not only by cancer diagnosis and treatment, but also by developmental stage, social role, and survivorship trajectory. Accordingly, the rise of this topic in keyword evolution highlights the need for developmentally informed and context-sensitive psychosocial care. Such care should not simply replicate models designed for older adults, but should address the developmental transitions and uncertainties specific to adolescence and young adulthood.

Digital health and AI-assisted approaches represent another important direction for future research and clinical translation. These tools may expand access to distress screening, remote monitoring, personalized support, and timely referral, particularly for patients with limited mobility or inadequate access to face-to-face psychosocial services [39]. In addition, physical activity interventions delivered through wearable devices have shown promise in reducing anxiety and depression among adolescents and young patients with cancer, although further studies are needed to confirm their effectiveness across different populations and care settings [40]. These findings indicate that future digital interventions should place greater emphasis on personalization, interactivity, sustained engagement, and adaptation to the needs of different patient groups. Artificial intelligence has further expanded the possibilities for early identification and targeted intervention. By integrating multidimensional patient data, AI-assisted screening may help identify individuals at high risk of psychological distress and support timely referral and intervention. Network analysis approaches are also increasingly being used to characterize the complex interrelationships among symptoms such as anxiety, depression, and distress, thereby helping to identify central symptoms and potential intervention targets. In addition, integrated platforms combining telemedicine and AI, such as OnkoRiskNET, have demonstrated potential in hereditary cancer risk management by improving access to remote genetic counseling, supporting multidisciplinary collaboration, and reducing psychological burden in patients and their families [41]. These developments suggest that AI may contribute not only to screening efficiency, but also to the broader integration of precision medicine and psychosocial care.

However, the expansion of digital and AI-enabled approaches also raises important challenges. First, digital tools often involve the collection, storage, and transmission of sensitive psychological and clinical data, making privacy protection and data security essential. Second, AI models may reproduce or amplify existing biases if training datasets are not sufficiently representative across age, sex, ethnicity, socioeconomic status, cancer type, disease stage, and healthcare setting. Such algorithmic bias may lead to unequal risk prediction or inappropriate referral recommendations for underrepresented groups [42,43]. Third, unequal digital access may limit the benefits of these tools. Patients with limited internet access, lower digital literacy, older age, financial constraints, or inadequate private space for remote consultation may be less able to use digital psychosocial services effectively [44]. Therefore, future research should address privacy protection, data security, algorithmic bias, unequal digital access, patient and clinician acceptability, and clinical governance. Digital and AI-enabled tools should be developed as adjuncts to clinician-led psychosocial care, with clear mechanisms for oversight, validation, accountability, and escalation when severe distress or suicide risk is detected. Future studies may also benefit from integrating psychological assessment with clinical indicators, symptom data, inflammatory or immune markers, and patient-reported outcomes, as well as from strengthening interdisciplinary collaboration among oncology, psychology, psychiatry, nursing, rehabilitation, palliative care, data science, and public health.

Overall, the findings suggest that research on cancer-related psychological distress is entering a more clinically relevant, interdisciplinary, and translational stage. Future studies should place greater emphasis on standardized screening, longitudinal monitoring, population-specific support, integrated psychosocial–somatic care, caregiver involvement, and responsible digital innovation. These directions may help promote the incorporation of psychological distress management into routine oncology practice and support more proactive, personalized, and patient-centered cancer care.

## 5. Strengths and Limitations

This study used bibliometric analysis to provide a broad overview of research trends, knowledge structures, collaboration patterns, emerging hotspots, and future directions in cancer-related psychological distress. By integrating data from WoSCC and Scopus and applying multiple bibliometric tools, including VOSviewer, CiteSpace, and bibliometrix, this study mapped the development of the field from multiple perspectives, including publication trends, country and institutional contributions, journal distribution, author collaboration, co-cited references, keyword co-occurrence, burst keywords, and thematic evolution.

Several limitations should be acknowledged. First, although WoSCC and Scopus were used as the primary bibliometric databases, other databases such as Embase, PsycINFO, CINAHL, and PubMed were not included in the final bibliometric dataset, which may have led to the omission of relevant studies. Second, only English-language publications were included, which may have introduced language bias and may partly explain the dominance of English-speaking countries, high-income countries, and highly indexed institutions. Third, database selection, citation practices, and indexing bias may have influenced the observed patterns of country, institution, journal, and author contributions. Publications from regions, institutions, or journals with lower international indexing visibility may have been underrepresented. Fourth, although duplicate removal and manual verification were performed, data cleaning and standardization in bibliometric research remain challenging. Variations in author names, institutional names, abbreviations, and keyword expressions may have affected the accuracy of collaboration networks and productivity analyses. Author and institution name ambiguity may be particularly difficult to eliminate completely, especially when different spelling formats, name changes, institutional mergers, or inconsistent affiliations are present across databases. Fifth, this study mainly used citation counts and related bibliometric indicators, but did not include normalized citation indicators adjusted by field, publication year, or document type. Therefore, citation impact should be interpreted cautiously, because older publications and papers from highly cited journals or research areas may have greater citation advantages. Sixth, the database search was conducted on 10 March 2025. Therefore, the findings reflect the state of the literature and citation data available at the time of the database search. Given the time interval between the database search and manuscript submission, more recent publications, citation changes, keyword shifts, and emerging topics may not have been captured. As a result, the findings may not fully reflect the most recent developments in this field.

Despite these limitations, this study provides a useful overview of the research landscape, thematic structure, and developmental trajectory of cancer-related psychological distress. The findings may help researchers and clinicians understand the evolution of this field, identify major contributors and emerging topics, and develop future studies focusing on standardized screening, survivorship care, caregiver support, digital health, and integrated psychosocial–somatic management.

## 6. Conclusions

This bibliometric analysis provides a comprehensive overview of global research on cancer-related psychological distress from 2015 to 2024. The findings reveal sustained growth in publication output and identify influential contributors, collaboration patterns, core themes, and emerging topics in this field. Research attention has increasingly shifted toward quality of life, distress screening, survivorship care, caregiver support, symptom burden, and digital health, suggesting that cancer-related psychological distress has become an important issue in supportive oncology and comprehensive cancer care.

These findings should be interpreted cautiously. As a bibliometric study, this work identifies research trends and thematic evolution but does not establish the clinical effectiveness or evidence quality of specific interventions. Future studies should strengthen standardized assessment, longitudinal follow-up, interdisciplinary collaboration, and population-specific psychosocial support. Digital and artificial intelligence-assisted approaches should be regarded as emerging areas that require further validation in terms of effectiveness, safety, equity, acceptability, and clinical governance before routine implementation.

## Figures and Tables

**Table 1:** Top 10 Countries by Publication Volume.

Country	Documents	Citations	Citations Per Paper	Total Link Strength
United States	2194	56,613	25.81	895
Australia	664	20,609	31.04	469
China	650	10,497	16.15	253
Germany	471	12,637	26.83	278
United Kingdom	464	14,015	30.21	555
Canada	411	10,847	26.39	361
Italy	376	7193	19.13	266
Netherlands	370	14,062	38.01	315
Japan	198	2409	12.17	79
Spain	192	3861	20.11	140

Note: “Citations per paper” refers to the mean number of citations per publication, calculated as total citations divided by the number of publications. Country attribution was based on author affiliation information. When a publication included authors from multiple countries, each country was counted in the country-level analysis.

**Table 2:** Top 10 Institutions by Publication Volume.

Organization	Documents	Citations	Citations Per Paper	Total Link Strength
University of Sydney	179	8035	44.89	652
Harvard Medical School	161	4342	26.97	587
University of Melbourne	140	3360	24.00	586
Memorial Sloan Kettering Cancer Center	136	4397	32.33	330
Duke University	133	2515	18.91	384
University of Toronto	131	4414	33.69	363
The University of Texas MD Anderson Cancer Center	121	2526	20.88	242
Dana-Farber Cancer Institute	116	2932	25.28	450
Radboud University Nijmegen	108	3113	28.82	281
Massachusetts General Hospital	102	2669	26.17	316

**Table 3:** Top 10 Journals by Publication Volume.

Journal Name	Documents	Citations	Citations Per Paper	IF	JCR
*Psycho-Oncology*	483	13,078	27.08	3.5	Q1
*Supportive Care in Cancer*	396	8056	20.34	3	Q1
*Frontiers in Psychology*	136	2310	16.99	2.9	Q1
*Cancer*	111	5828	52.50	5.1	Q1
*European Journal of Cancer Care*	107	2143	20.03	1.9	Q2
*Palliative & Supportive Care*	104	1677	16.13	2.1	Q3
*Cancer Nursing*	100	1893	18.93	2.5	Q1
*European Journal of Oncology Nursing*	98	1906	19.45	2.7	Q1
*Journal of Psychosocial Oncology*	96	1312	13.67	1.5	Q4
*BMJ Open*	85	827	9.73	2.3	Q2

**Table 4:** Top 10 Journals by Citation Count.

Journal Name	Citations	Documents	Citations Per Paper	IF	JCR
*Psycho-Oncology*	13,078	483	27.08	3.5	Q1
*Supportive Care in Cancer*	8056	396	20.34	3	Q1
*Cancer*	5828	111	52.50	5.1	Q1
*Journal of Pain and Symptom Management*	2433	84	28.96	3.5	Q1
*Journal of Clinical Oncology*	2426	30	80.87	43.4	Q1
*Frontiers in Psychology*	2310	136	16.99	2.9	Q1
*European Journal of Cancer Care*	2143	107	20.03	1.9	Q2
*BMC Cancer*	2126	75	28.35	3.4	Q2
*Journal of Cancer Survivorship*	1984	81	24.49	2.9	Q1
*PLoS ONE*	1950	82	23.78	2.6	Q2

Note: “Citations per paper” refers to the mean number of citations per publication. Journal quartiles were classified according to the 2024 Journal Citation Reports (JCR). When a journal was listed in multiple JCR categories, the category most relevant to oncology, psychology, palliative care, nursing, or supportive care was used for quartile classification.

**Table 5:** Top 10 Authors by Publication Volume.

Author	Documents	Citations	Citations Per Paper
Anja Mehnert	58	1651	28.47
Areej El-Jawahri	53	1385	26.13
Eduardo Bruera	38	1042	27.42
Phyllis Butow	34	1465	43.09
Judith B. Prins	34	754	22.18
Caterina Calderon	30	404	13.47
Suzanne K. Chambers	29	1167	40.24
Leslie L. Robison	28	882	31.50
Paula Jimenez-Fonseca	28	397	14.18
Kevin R. Krull	26	616	23.69

**Table 6:** Top 10 Authors by Citation Count.

Author	Documents	Citations	Citations Per Paper
Anja Mehnert	58	1651	28.47
Phyllis Butow	34	1465	43.09
David Cella	15	1428	95.20
Stephen Ross	5	1422	284.40
Areej El-Jawahri	53	1385	26.13
Suzanne K. Chambers	29	1167	40.24
Jennifer S. Temel	25	1096	43.84
Joseph A. Greer	25	1044	41.76
Eduardo Bruera	38	1042	27.42
Leslie L. Robison	28	882	31.50

## Data Availability

The bibliographic dataset analyzed in this study was created from records retrieved from the Web of Science Core Collection and Scopus according to the search strategy described in the Methods and Supplementary Materials. The raw records exported from the Web of Science Core Collection and Scopus are subject to the access and licensing policies of the respective databases. Additional analysis files or code used for bibliometric visualization and statistical processing are available from the corresponding authors upon reasonable request.

## Supplementary Materials

The following supporting information can be downloaded at: https://www.mdpi.com/article/10.3390/healthcare14142195/s1, Supplementary Material S1. Detailed search strategies. Figure S1: Reference co-citation network in research on cancer-related psychological distress. Figure S2: Research Hotspot Trends related to cancer-related psychological distress.

## References

[1] Bray F., Laversanne M., Sung H., Ferlay J., Siegel R.L., Soerjomataram I., Jemal A. (2024). Global cancer statistics 2022: GLOBOCAN estimates of incidence and mortality worldwide for 36 cancers in 185 countries. CA Cancer J. Clin..

[2] Getie A., Ayalneh M., Bimerew M. (2025). Global prevalence and determinant factors of pain, depression, and anxiety among cancer patients: An umbrella review of systematic reviews and meta-analyses. BMC Psychiatry.

[3] Riba M.B., Donovan K.A., Andersen B., Braun I., Breitbart W.S., Brewer B.W., Buchmann L.O., Clark M.M., Collins M., Corbett C. (2019). Distress Management, Version 3.2019, NCCN Clinical Practice Guidelines in Oncology. J. Natl. Compr. Cancer Netw..

[4] Bergerot C.D., Bergerot P.G., Philip E.J., Ferrari R., Peixoto R.M., Crane T.E., Schmitz K.H., Soto-Perez-de-Celis E. (2024). Enhancing Cancer Supportive Care: Integrating Psychosocial Support, Nutrition, and Physical Activity Using Telehealth Solutions. JCO Glob. Oncol..

[5] Carlson L.E. (2023). Psychosocial and Integrative Oncology: Interventions Across the Disease Trajectory. Annu. Rev. Psychol..

[6] Zeng Y., Hu C.H., Li Y.Z., Zhou J.S., Wang S.X., Liu M.D., Qiu Z.H., Deng C., Ma F., Xia C.F. (2024). Association between pretreatment emotional distress and immune checkpoint inhibitor response in non-small-cell lung cancer. Nat. Med..

[7] Ikhile D., Ford E., Glass D., Gremesty G., van Marwijk H. (2024). A systematic review of risk factors associated with depression and anxiety in cancer patients. PLoS ONE.

[8] Patel P.G., Dagli C., Al-Antary N., Nair M., Babatunde O.A., Osazuwa-Peters N., Satheeshkumar P.S., Boakye E.A. (2025). The Association Between Psychological Distress, Emergency Room Visits, and All-Cause Mortality Among Colorectal Cancer Survivors. Cancer Med..

[9] Thakerar A., Simadri K., Alexander M., Fullerton S. (2020). Paroxetine for the treatment of intractable and persistent cough in patients diagnosed with cancer. J. Oncol. Pharm. Pract..

[10] Grassi L. (2020). Psychiatric and psychosocial implications in cancer care: The agenda of psycho-oncology. Epidemiol. Psychiatr. Sci..

[11] McCarter K., Britton B., Baker A., Halpin S., Beck A., Carter G., Wratten C., Bauer J., Booth D., Forbes E. (2015). Interventions to improve screening and appropriate referral of patients with cancer for distress: Systematic review protocol. BMJ Open.

[12] Powell K.R., Peterson S.R. (2017). Coverage and quality: A comparison of Web of Science and Scopus databases for reporting faculty nursing publication metrics. Nurs. Outlook.

[13] Asubiaro T.V. (2023). Sub-Saharan Africa’s biomedical journal coverage in scholarly databases: A comparison of Web of Science, Scopus, EMBASE, MEDLINE, African Index Medicus, and African Journals Online. J. Med. Libr. Assoc..

[14] Deshields T.L., Potter P., Olsen S., Liu J. (2014). The persistence of symptom burden: Symptom experience and quality of life of cancer patients across one year. Support. Care Cancer.

[15] Wu H.S., Harden J.K. (2015). Symptom burden and quality of life in survivorship: A review of the literature. Cancer Nurs..

[16] Correa-Morales J.E., Cuellar-Valencia L., Mantilla-Manosalva N., Quintero-Muñoz E., Iriarte-Aristizábal M.F., Giraldo-Moreno S., Rodríguez-Campos L.F. (2023). Cancer and Non-cancer Fatigue Treated with Bupropion: A Systematic Review. J. Pain Symptom Manag..

[17] Jim H.S.L., Hoogland A.I., Han H.S., Culakova E., Heckler C., Janelsins M., Williams G.C., Bower J., Cole S., Desta Z. (2020). A randomized placebo-controlled trial of bupropion for Cancer-related fatigue: Study design and procedures. Contemp. Clin. Trials.

[18] Ajo R., Segura A., Inda M.M., Planelles B., Martínez L., Ferrández G., Sánchez A., César M., Peiró A.M. (2016). Opioids Increase Sexual Dysfunction in Patients with Non-Cancer Pain. J. Sex. Med..

[19] Salata B., Kluczna A., Dzierżanowski T. (2022). Opioid-Induced Sexual Dysfunction in Cancer Patients. Cancers.

[20] Yee A., Loh H.S., Ong T.A., Ng C.G., Sulaiman A.H. (2018). Randomized, Double-Blind, Parallel-Group, Placebo-Controlled Trial of Bupropion as Treatment for Methadone-Emergent Sexual Dysfunction in Men. Am. J. Men’s Health.

[21] Tatari F., Farnia V., Nasiri R.F., Najafi F. (2010). Trazodone in methandone induced erectile dysfunction. Iran. J. Psychiatry.

[22] Ramli F.F., Azizi M.H., Syed Hashim S.A. (2021). Treatments of Sexual Dysfunction in Opioid Substitution Therapy Patients: A Systematic Review and Meta-Analysis. Int. J. Med. Sci..

[23] Molassiotis A., Cheng H.L., Lopez V., Au J.S.K., Chan A., Bandla A., Leung K.T., Li Y.C., Wong K.H., Suen L.K.P. (2019). Are we mis-estimating chemotherapy-induced peripheral neuropathy? Analysis of assessment methodologies from a prospective, multinational, longitudinal cohort study of patients receiving neurotoxic chemotherapy. BMC Cancer.

[24] Zajączkowska R., Kocot-Kępska M., Leppert W., Wrzosek A., Mika J., Wordliczek J. (2019). Mechanisms of Chemotherapy-Induced Peripheral Neuropathy. Int. J. Mol. Sci..

[25] Sandhya L., Devi Sreenivasan N., Goenka L., Dubashi B., Kayal S., Solaiappan M., Govindarajalou R., Kt H., Ganesan P. (2023). Randomized Double-Blind Placebo-Controlled Study of Olanzapine for Chemotherapy-Related Anorexia in Patients with Locally Advanced or Metastatic Gastric, Hepatopancreaticobiliary, and Lung Cancer. J. Clin. Oncol..

[26] Sakai H., Tsurutani J., Ozaki Y., Ishiguro H., Nozawa K., Yamanaka T., Aogi K., Matsumoto K., Iwasa T., Tokiwa M. (2025). A randomized, double-blind, placebo-controlled phase II study of olanzapine-based prophylactic antiemetic therapy for delayed and persistent nausea and vomiting in patients with HER2-positive or HER2-low breast cancer treated with trastuzumab deruxtecan: ERICA study (WJOG14320B). Ann. Oncol..

[27] Ye Z.J., Qiu H.Z., Li P.F., Liang M.Z., Zhu Y.F., Zeng Z., Hu G.Y., Wang S.N., Quan X.M. (2017). Predicting changes in quality of life and emotional distress in Chinese patients with lung, gastric, and colon-rectal cancer diagnoses: The role of psychological resilience. Psychooncology.

[28] Huang X.Y., Qian D. (2025). Effect of multidisciplinary team collaborative nursing on wound healing and psychological symptoms in postoperative patients with gastrointestinal tumors. World J. Gastrointest. Oncol..

[29] Oliveira F.F.B., de Barros Silva P.G., de Sant’Ana R.O., de Albuquerque C.G.P., Bezerra M.J.B., Wong D.V.T., da Silveira Bitencourt F., de Lima Silva-Fernandes I.J. (2021). Lima MVA: How does genetic testing influence anxiety, depression, and quality of life? A hereditary breast and ovarian cancer syndrome suspects trial. Support. Care Cancer.

[30] Zhang W., Ning X., Chen H., Li X., Li J., Yang X. (2025). Effects of Non-Pharmacological Interventions on Psychological Distress in Patients with Malignant Tumors: A Systematic Review and Network Meta-Analysis. Worldviews Evid. Based Nurs..

[31] van Hof K.S., Hoesseini A., Dorr M.C., Verdonck-de Leeuw I.M., Jansen F., Leemans C.R., Takes R.P., Terhaard C.H.J., de Jong R.J.B., Sewnaik A. (2023). Unmet supportive care needs among informal caregivers of patients with head and neck cancer in the first 2 years after diagnosis and treatment: A prospective cohort study. Support. Care Cancer.

[32] Adjei Boakye E., Polednik K.M., Deshields T.L., Sharma A., Molina Y., Schapira L., Barnes J.M., Osazuwa-Peters N. (2022). Emotional distress among survivors of adolescent and young adult cancer or adult cancer. Ann. Epidemiol..

[33] Osmani V., Hörner L., Klug S.J., Tanaka L.F. (2023). Prevalence and risk of psychological distress, anxiety and depression in adolescent and young adult (AYA) cancer survivors: A systematic review and meta-analysis. Cancer Med..

[34] Liu C., Yuan Y.C., Guo M.N., Xin Z., Chen G.J., Ding N., Zheng J.P., Zang B., Yang J.K. (2024). Rising incidence of obesity-related cancers among younger adults in China: A population-based analysis (2007–2021). Med.

[35] Qian J., Qi S., Hu R., Zhang M., Chen Z., Ding Z. (2025). Trends in the disease burden of thyroid. cancer among adolescents and young adults: A comparative study of China and global estimates (1990–2021). PLoS ONE.

[36] Matsuo K., Duval C.J., Nanton B.A., Yao J.A., Yu E., Pino C., Wright J.D. (2024). Suicide Deaths Among Adolescent and Young Adult Patients with Cancer. JAMA Netw. Open.

[37] Michalek I.M., Caetano Dos Santos F.L., Wojciechowska U., Didkowska J. (2023). Suicide risk among adolescents and young adults after cancer diagnosis: Analysis of 34 cancer groups from 2009 to 2019. J. Cancer Surviv..

[38] McGrady M.E., Willard V.W., Williams A.M., Brinkman T.M. (2024). Psychological Outcomes in Adolescent and Young Adult Cancer Survivors. J. Clin. Oncol..

[39] Vrancken Peeters N., Georgopoulou S., Kulakowski R., Hainsworth E., Lidington E., McGrath S.E., Noble J., Azarang L., Husson O., Cruickshank S. (2025). Effect of a digital tool on breast cancer specific health-related quality of life and psychological distress: Secondary results from the ADAPT study. Support. Care Cancer.

[40] Li L., Wang L., Duan Y., Xiao P., Zhou Y., Luo X., Liu X., Xie J., Cheng A.S.K. (2023). Intelligent physical activity versus modified behavioral activation in adolescent and young adult cancer patients with psychological distress: A randomized, controlled pilot trial. Cancer Med..

[41] Tecklenburg J., Vajen B., Morlot S., Anders P., Memenga P., Link E., Baumann E., Wölffling S., Schröck E., Bergmann A.K. (2022). OnkoRiskNET: A multicenter, interdisciplinary, telemedicine-based model to improve care for patients with a genetic tumor risk syndrome. BMC Health Serv. Res..

[42] Norori N., Hu Q., Aellen F.M., Faraci F.D., Tzovara A. (2021). Addressing bias in big data and AI for health care: A call for open science. Patterns.

[43] Yang Y., Lin M., Zhao H., Peng Y., Huang F., Lu Z. (2024). A survey of recent methods for addressing AI fairness and bias in biomedicine. J. Biomed. Inform..

[44] Richardson S., Lawrence K., Schoenthaler A.M., Mann D. (2022). A framework for digital health equity. npj Digit. Med..

