# Supplementary material for: Cancer-Related Psychological Distress over the Past Decade: A Bibliometric Analysis of Research Trends, Hotspots, and Emerging Areas
Source: Healthcare (Basel). 2026 Jul 20;14(14):2195. doi: 10.3390/healthcare14142195 (PMC13409850; doi:10.3390/healthcare14142195)
Supplement: Supplementary file 1 [file healthcare-14-02195-s001.zip › healthcare-4319960-supplementary.pdf]

Supplementary Material S1. Detailed search strategies

1.The search strategy

The search strategy used in WoSCC was as follows:

(((TS=("psychological distress" OR "distress thermometer" OR "emotional distress")) AND TS=(neoplas OR cancer OR oncolog* OR tumor* OR tumour* OR carcinoma* OR malignan*)) AND DOP=(2015-01-01/2024-12-31) AND DT=(Article OR Review) AND LA=(English))

The search strategy used in Scopus was as follows:

TITLE-ABS-KEY ("psychological distress" OR "distress thermometer" OR "emotional distress") AND TITLE-ABS-KEY (neoplas* OR cancer OR oncolog* OR tumor* OR tumour* OR carcinoma* OR malignan*) AND PUBYEAR > 2014 AND PUBYEAR < 2025 AND (LIMIT-TO (DOCTYPE , "ar") OR LIMIT-TO (DOCTYPE , "re")) AND (LIMIT-TO (LANGUAGE , "English"))

2. Eligibility Criteria and Literature Selection

The inclusion criteria were as follows:

publications focused on cancer-related psychological distress or closely related topics;

records indexed in WoSCC or Scopus within the predefined period from 2015 to 2024;

publications written in English;

document types in WoSCC and Scopus limited to articles and reviews.

The exclusion criteria were as follows:

studies not relevant to cancer-related psychological distress after title and abstract screening;

duplicate records retrieved from multiple databases;

document types other than articles and reviews;

records with incomplete bibliographic information that were unsuitable for bibliometric analysis.

All records retrieved from WoSCC and Scopus were exported and imported into EndNote X9 for reference management. Duplicate records were identified using the software’s automatic duplicate detection function and then manually verified.

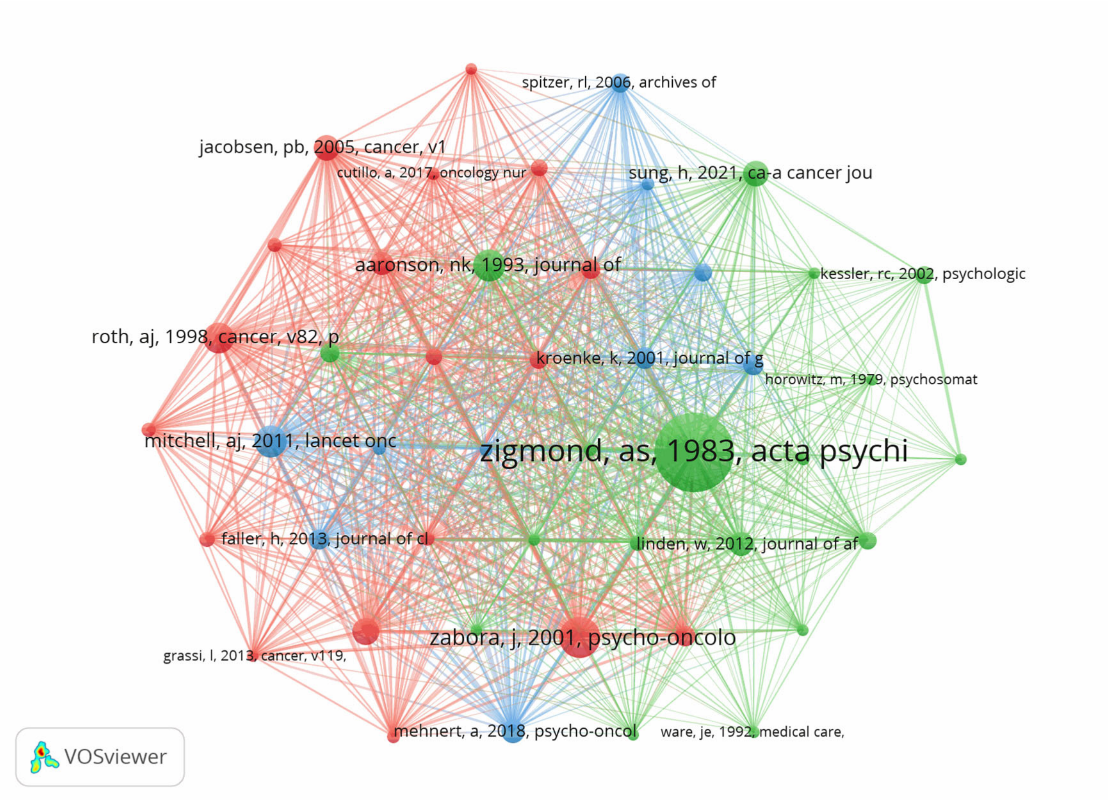

Figure S1. Reference co-citation network in research on cancer-related psychological distress. Each node represents a cited reference, and the node size is proportional to its co-citation frequency. The connecting lines represent co-citation relationships between references, with thicker lines indicating stronger relationships. Different colors represent distinct reference clusters identified by VOSviewer.

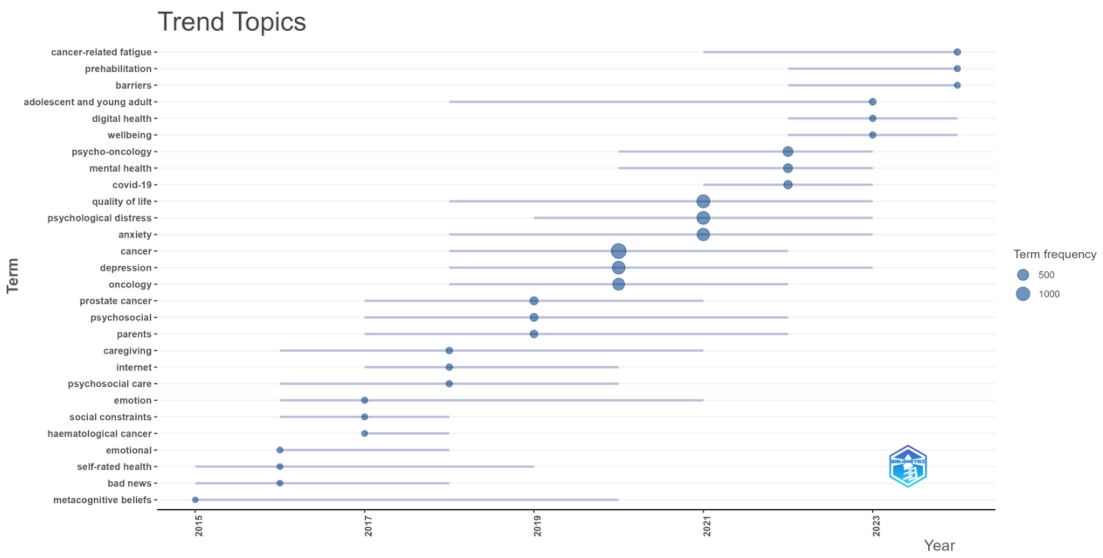

Figure S2. Research Hotspot Trends related to cancer-related psychological distress. Each horizontal line indicates the time span during which a research topic was active. The position of each bubble represents the corresponding year in the trend analysis, and the bubble size is proportional to the frequency of the term.
